# Patient-derived hiPSC neurons with heterozygous *CNTNAP2* deletions display altered neuronal gene expression and network activity

**DOI:** 10.1038/s41537-017-0033-5

**Published:** 2017-10-02

**Authors:** Erin Flaherty, Rania M. Deranieh, Elena Artimovich, Inkyu S. Lee, Arthur J. Siegel, Deborah L. Levy, Michael W. Nestor, Kristen J. Brennand

**Affiliations:** 1 Departments of Neuroscience, Icahn School of Medicine at Mount Sinai, New York, NY 10029 USA; 2 Friedman Brain Institute, Icahn School of Medicine at Mount Sinai, New York, NY 10029 USA; 3 Hussman Institute for Autism, 801W. Baltimore St., Baltimore, MD 21201 USA; 4 Internal Medicine Department, McLean Hospital, Belmont, MA 02478 USA; 5 Psychology Research Laboratory, McLean Hospital, Belmont, MA 02478 USA; 6 Departments of Psychiatry, Icahn School of Medicine at Mount Sinai, New York, NY 10029 USA

## Abstract

Variants in *CNTNAP2*, a member of the neurexin family of genes that function as cell adhesion molecules, have been associated with multiple neuropsychiatric conditions such as schizophrenia, autism spectrum disorder and intellectual disability; animal studies indicate a role for *CNTNAP2* in axon guidance, dendritic arborization and synaptogenesis. We previously reprogrammed fibroblasts from a family trio consisting of two carriers of heterozygous intragenic *CNTNAP2* deletions into human induced pluripotent stem cells (hiPSCs) and described decreased migration in the neural progenitor cells (NPCs) differentiated from the affected *CNTNAP2* carrier in this trio. Here, we report the effect of this heterozygous intragenic deletion in *CNTNAP2* on global gene expression and neuronal activity in the same cohort. Our findings suggest that heterozygous *CNTNAP2* deletions affect genes involved in neuronal development and neuronal activity; however, these data reflect only one family trio and therefore more deletion carriers, with a variety of genetic backgrounds, will be needed to understand the molecular mechanisms underlying *CNTNAP2* deletions.

## Short Report

The shared genetic architecture underlying neuropsychiatric disorders implicates common molecular mechanisms.^1^ For example, while homozygous null mutations in *CNTNAP2* lead to cortical dysplasia-focal epilepsy syndrome,^2, 3^ heterozygous intragenic deletions are associated with schizophrenia, intellectual disability, language deficits, seizures, and autism traits.^4^ Critically, *CNTNAP2* variants are not completely penetrant.^2, 5^ Animal studies indicate a role for *CNTNAP2* in axon guidance, dendritic arborization, and synaptogenesis.^6–8^

We obtained fibroblast samples from a family trio with two carriers of heterozygous intragenic *CNTNAP2* deletions, one affected and one unaffected, and an unaffected non-carrier control (Table 1). The *CNTNAP2* carriers display discordant clinical phenotypes; the daughter (DL7078) presented with schizo-affective disorder (depressed subtype) while the father (DL8735) was neurotypical.^9^ We previously used sendai viral vectors to reprogram fibroblasts from this trio into hiPSCs that were then differentiated via dual-SMAD induction into NPCs and neurons. We characterized decreased migration in NPCs and allele-biased expression of the mutant *CNTNAP2* transcript by qPCR in neurons from the affected *CNTNAP2* carrier in this trio.^9^ Here, we report the effect of this heterozygous intragenic deletion in *CNTNAP2* on global gene expression and neuronal activity in this same cohort.

**Table 1:** Available clinical information on hiPSC donors

Patient ID	Source	hiPSC Line	Family	Sex	Dx	Age of Onset	IQ	Clozapine Response	Family History
DL7078	McLeans	7078 hiPSC#B	Proband	F	SA	18.9	100	Y	–
DL8735	McLeans	8735 hiPSC#H	Father	M	Control	n/a	120	n/a	SA
DL5535	McLeans	5535 hiPSC#2	Mother	F	Control	n/a	95	n/a	SA
NSB3113	NIH	3113 hiPSC#1	Non-relative	F	Control	n/a	123	n/a	n/a

**Fig. 1:**
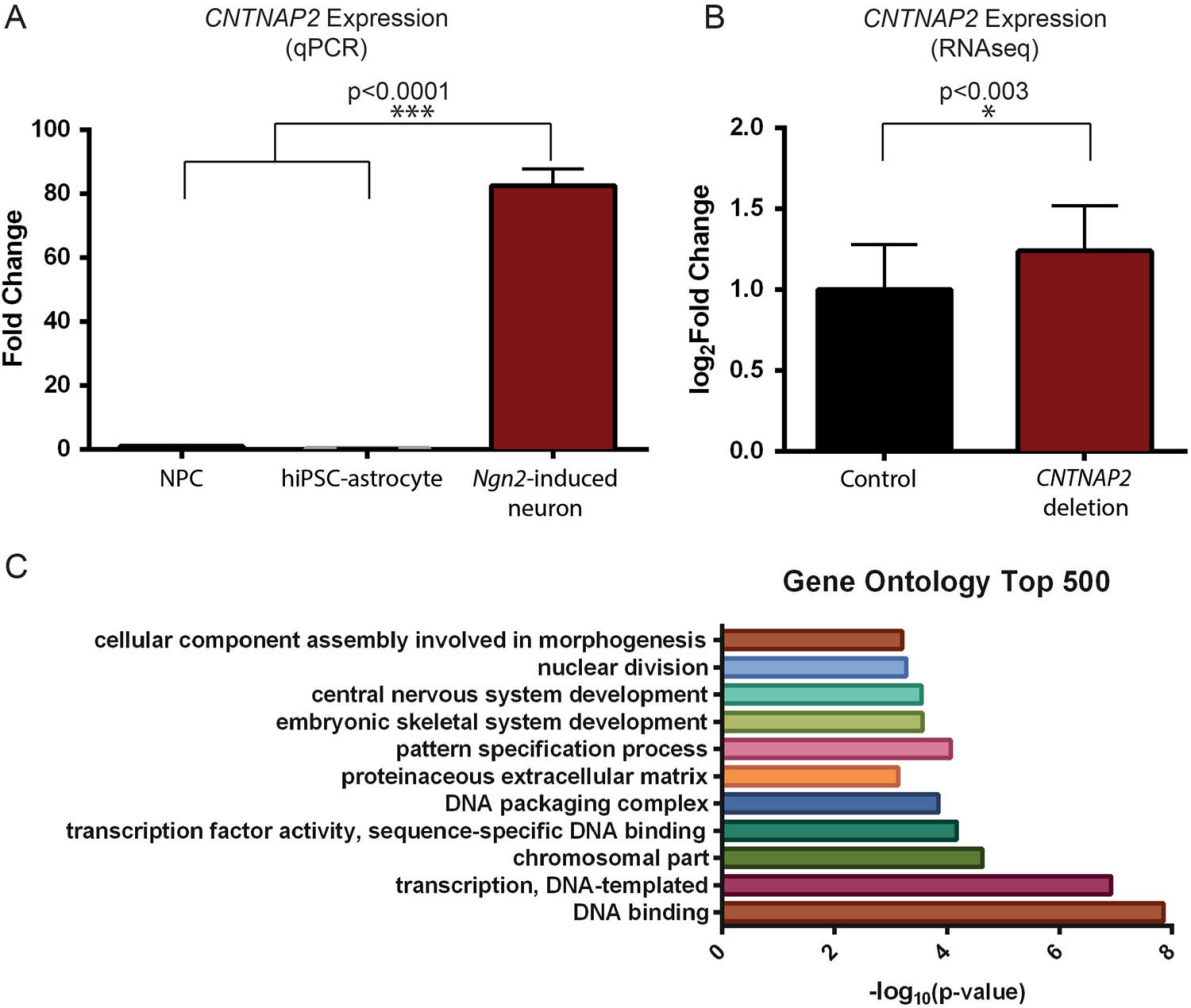
*CNTNAP2* expression in excitatory neurons induced from family trio. **a** By qPCR, *CNTNAP2* expression is significantly increased in *Ngn2*-induced neurons compared to neural progenitor cells (NPCs) and hiPSC-derived astrocytes. Data reflects biological triplicate samples from one hiPSC line derived from each of three healthy controls (mean +/− s.e.m). **b** RNA sequencing data shows total *CNTNAP2* expression is increased in deletion carriers compared with non-carrier control (mean +/− s.e.m). **c** Genes differentially expressed in *CNTNAP2* deletion carriers are enriched for genes involved in DNA binding and central nervous system development. * *p* < 0.05, *** *p* < 0.001

*CNTNAP2* is highly expressed in *Ngn2*-induced neurons, a population of nearly pure excitatory neurons,^10^ relative to hiPSC-derived NPCs^11^ and hiPSC-astrocytes^12^ (qPCR FC = 82.5, *p* < 0.0001, ANOVA with Tukey’s Post-Hoc) (Fig. 1a). RNA was harvested after 21 days of *Ngn2*-induction. The New York Genome Center prepared RNAseq libraries using the Kapa Total 350 bp kit, followed by 2 × 125 bp Illumina RNA sequencing to a read depth of 40 M reads per sample on the HiSeq 2500.

We queried the expression of *CNTNAP2* in *Ngn2*-induced neurons from each member of this family trio, hypothesizing that heterozygous intragenic deletions may affect the expression of *CNTNAP2*. Surprisingly, overall *CNTNAP2* expression was increased in the *CNTNAP2* deletion carriers compared with the non-carrier mother (log_2_FC = 1.24, padj = 0.003) (Fig. 1b).

Differential expression analysis was performed using DESeq2^13^ and the top 500 differentially expressed genes were used to perform gene ontology using DAVID^14, 15^ (SI Table 1). The most significant subset of genes mapped to terms relating to DNA binding and central nervous system (CNS) development (FC = 1.8, *p* < 0.00001 and FC = 1.9, *p* = 0.0003) (Fig. 1c; SI Table 2). Within the gene subset involved in CNS development, there are some interesting candidate genes such as *CNTN6* and *CNTN4*, which are involved in regulating cell surface interactions during nervous system development and are also thought to be important in synaptogenesis (SI Table 3 **)**.

**Fig. 2:**
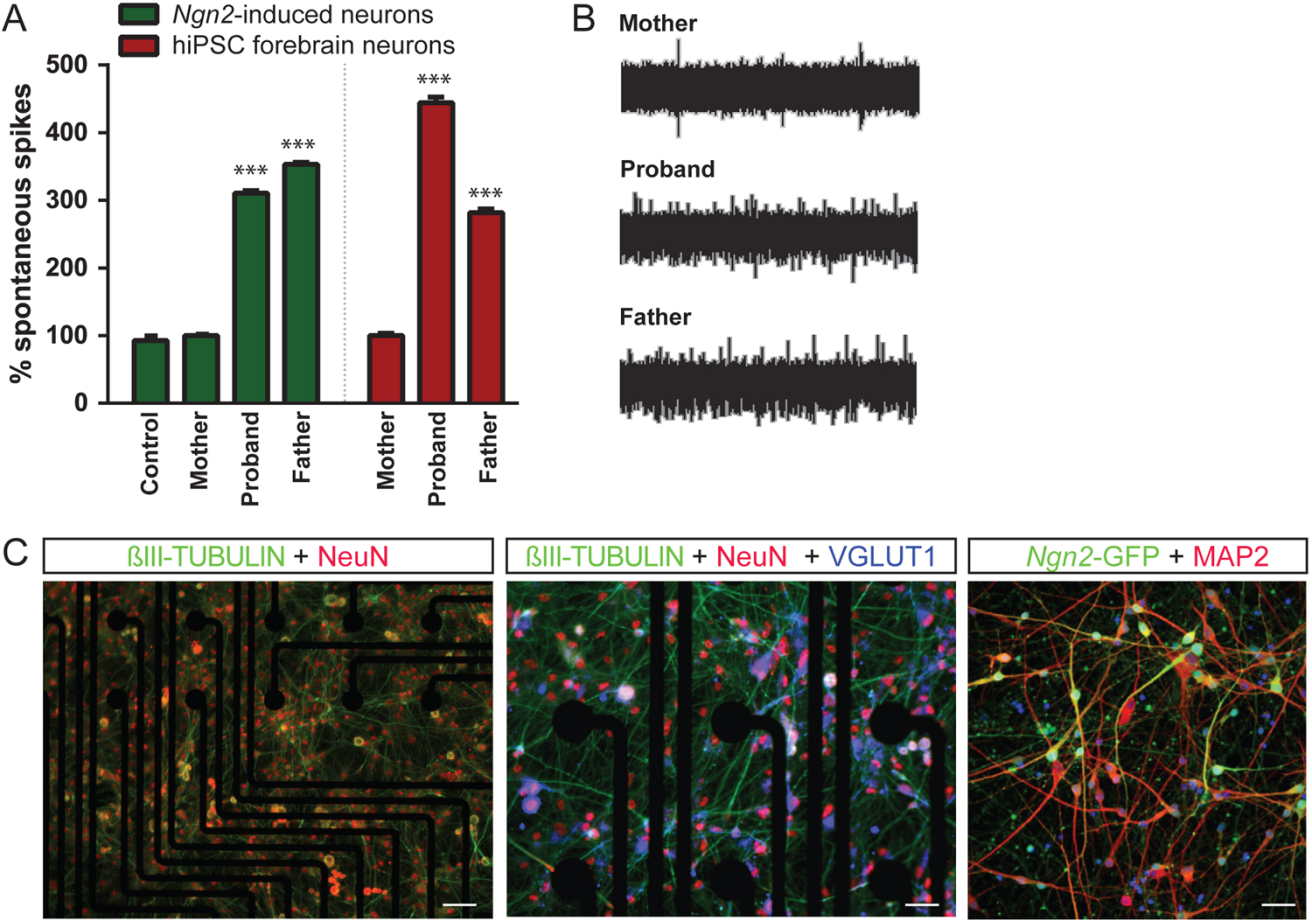
hiPSC-derived neurons from *CNTNAP2* carriers show increased neuronal activity compared to control. **a** Summary data of population-wide MEA spike frequency of individuals in the family trio in both the *Ngn2*-induced neurons (green) and hiPSC-derived forebrain mixed neuronal populations (red) reveal significantly increased synaptic activity in the *CNTNAP2* deletion carriers. *** *p* < 0.001 **b** Representative traces of population-wide neuronal activity measured by MEA. **c** Representative staining of directed differentiation neurons (left and center) at Day28 for general neuronal markers on MEA plates demonstrate normal expression of TUJ1 (green), NEUN (red); (left, 100X), as well as VGLUT1 (blue); (center, 200X)) (s.b. left = 100 μM; right = 50 μM). Representative staining of *Ngn2-*induced neurons (right) for MAP2 and, as well as expression of *hNgn2*-eGFP-Puro^R^ (addgene #79823) at Day21 (right, 200X) (s.b. = 40 μM)

Given the differences in gene expression of critical neuronal and synaptic genes, we applied an Axion multi-electrode array (MEA) (see similar applications to Amyotrophic Lateral Sclerosis^16^ and Parkinson’s disease^17^) to record population-wide neuronal activity under conditions similar to those used in our RNAseq analyzes. 21-day-old *Ngn2*-induced neurons from both the affected (daughter, DL7078) and unaffected (father, DL8735) *CNTNAP2* deletion carriers showed significantly increased spontaneous network level activity (an increase of 210 and 253%, respectively) relative to the non-carrier (mother, DL5535) and an unaffected unrelated control (female, NSB3113) (*N* = 12 wells/condition; *p* < 0.001, ANOVA with Tukey’s Post-Hoc) (Fig. 2a). These results were confirmed in hiPSC-derived forebrain neuron populations, which are comprised of a mixture of mature and immature excitatory neurons, inhibitory neurons and astrocytes, with the majority of cells presumed to be excitatory neurons.^18, 19^ Here, NPCs were seeded (65,000 cells/mL) onto 12-well MEA plates and differentiated for 28 days; hiPSC-derived neurons from the affected and unaffected *CNTNAP2* deletion carriers showed significantly increased spontaneous population wide neuronal activity relative to the non-carrier mother (increases of 344 and 182% relative to the non-carrier mother; *N* = 12 wells/condition; *p* < 0.001, ANOVA with Tukey’s Post-Hoc) (Fig. 2a, b). All measurements were performed as biological triplicates for each hiPSC line and averaged across experiments. In both populations, neurons robustly stained for neuronal markers βIII-TUBULIN, NeuN, and MAP2 **(**representative images in Fig. 2c).

Our genetic analyzes and functional assays together show that heterozygous deletion of *CNTNAP2* may impact neuronal activity. The significant increase in spontaneous spiking activity in the unaffected carrier father and carrier daughter may underlie aspects of the aberrant behavior displayed by the proband. Additionally, this alteration in spike activity may in part explain observations of disrupted neuronal synchrony in *CNTNAP2-null* mice.^6^

Here we demonstrated that hiPSC-derived neurons from individuals with heterozygous intragenic deletions in *CNTNAP2* display differential expression of genes involved in synaptic transmission and altered neuronal activity, consistent with reports of disrupted cortical neuronal activity in *CNTNAP2-null* mice,^6^ and potentially independent of clinical outcome. Our report reflects results from just one family trio; a greater variety of disease-associated *CNTNAP2* mutations, on an array of genetic backgrounds, will be needed to understand the full breath of genotype-phenotype relationships with respect to *CNTNAP2*.^18, 20–22^

### Ethical approval

The methods were performed in accordance with relevant guidelines and regulations and approved by the McLean Hospital Institutional Review Board. Participants were subject to informed and written consent.

### Data availability

All case and control hiPSCs, which have been mycoplasma tested, will be deposited with the NIMH Center For Collaborative Studies Of Mental Disorders At RUCDR. RNAseq data for *CNTNAP2* carrier and non-carrier neurons (GEO GSE102838) have been deposited at the GeneExpression Omnibus (GEO) repository. Antibodies used in this study are: βIII-TUBULIN (1:500; Chicken; Biolegend; 801201), NeuN(1:100; Rabbit; Abcam; ab104225), MAP2 (1:500; Chicken; Abcam; ab5392).

## Electronic supplementary material

SI Tables 1-3
